# 17**β**-Estradiol counteracts pathological microtubule remodeling to enhance right ventricular function in preclinical models

**DOI:** 10.1172/JCI201385

**Published:** 2026-05-07

**Authors:** Ryan A. Moon, Rafael Sobrano Fais, Minwoo Kim, Neal T. Vogel, Jenna B. Mendelson, Lynn M. Hartweck, John P. Carney, Melissa K. Gardner, Sally E. Prins, Sasha Z. Prisco, Tim Lahm, Kurt W. Prins

**Affiliations:** 1 Lillehei Heart Institute, Cardiovascular Division, University of Minnesota, Minneapolis, Minnesota, USA.; 2 Division of Pulmonary, Critical Care, and Sleep Medicine, Department of Medicine, National Jewish Health, Denver, Colorado, USA.; 3 Division of Pulmonary, Allergy and Critical Care Medicine, University of Colorado Anschutz Medical Center, Aurora, Colorado, USA.; 4 Rocky Mountain Regional VA Medical Center, Aurora, Colorado, USA.; 5 Department of Integrative Biology and Physiology;; 6 Experimental Surgical Services, Department of Surgery; and; 7 Department of Genetics, Cell Biology, and Development, University of Minnesota, Minneapolis, Minnesota, USA.; 8 Gazes Cardiac Research Institute, Division of Cardiology, Department of Medicine, Medical University of South Carolina, Charleston, South Carolina, USA.

**Keywords:** Cardiology, Cell biology, Cytoskeleton

## Abstract

Estrogen exerts cardioprotective effects via microtubule regulation.

**To the Editor:** Right ventricular (RV) cardiomyocyte microtubule remodeling occurs in pulmonary hypertension, and heightened microtubule density promotes cardiomyocyte dysfunction (1). The microtubule cytoskeleton regulates myocyte hypertrophy, nuclear remodeling, t-tubule structure, and the proper localization of the gap junction protein, connexin-43 (1). We demonstrated 17β-estradiol modulates microtubule dynamics in vitro through a tip interaction that mimics colchicine’s microtubule-destabilizing effects (2). However, 17β-estradiol’s effects on the RV microtubule regulation in vivo are unknown. This question may have clinical implications because females have greater RV reserve than men in multiple cardiovascular conditions (3), and this may be related to 17β-estradiol’s effects on microtubule regulation.

In vitro, 17β-estradiol directly slowed microtubule polymerization rates (Figure 1A). Furthermore, 17β-estradiol blunted endothelin-mediated total and detyrosinated microtubule densification in induced pluripotent stem cell–cardiomyocytes (iPSC-CMs) (Figure 1, B and C).

Then, we probed the physiological effects of 17β-estradiol in a translational approach by treating male rats with 17β-estradiol for 2 weeks starting 2 weeks after pulmonary artery banding (PAB). Echocardiographic analysis revealed 17β-estradiol increased tricuspid plane annular systolic excursion (TAPSE) and RV free wall thickening (Figure 1D). RV systolic pressure was equivalently elevated in PAB-Vehicle and PAB-E2 animals, but estimated RV–pulmonary artery coupling was significantly enhanced by 17β-estradiol (Figure 1D).

Next, we determined how 17β-estradiol treatment altered multiple RV cardiomyocyte microtubule phenotypes. First, 17β-estradiol partially mitigated excess microtubule density (Figure 1E). Furthermore, 17β-estradiol attenuated both cardiomyocyte and cardiomyocyte nuclear hypertrophy (Figure 1F). Finally, 17β-estradiol improved t-tubule structural integrity and prevented the lateral membrane mislocalization of connexin-43 (Figure 1G). Importantly, 17β-estradiol did not impact desmin localization patterns (Supplemental Figure 1; supplemental material available online with this article; https://doi.org/10.1172/JCI201385DS1), implying its beneficial effects on cardiomyocyte morphology were predominately mediated through microtubule network modulation.

Finally, we examined how endogenous and exogenous 17β-estradiol impacted microtubule phenotypes and RV functional reserve in female monocrotaline rats. Monocrotaline females exhibited only slight increases in RV tubulin abundances (Supplemental Figure 2). Oophorectomy did not substantially impact tubulin regulation, but exogenous 17β-estradiol reduced levels of tubulin in RV extracts (Supplemental Figure 2). In contrast with males, RV cardiomyocyte hypertrophy in female rodents was less tightly associated with microtubule regulation; however, exogenous estrogen blunted cardiomyocyte hypertrophy (Supplemental Figure 2). To assess RV functional reserve, we performed Langendorff measurements of RV contractility. With increasing RV dilation and at a matched RV end-diastolic pressure, monocrotaline oophorectomized females failed to augment RV contractile force, a deficit rescued by exogenous 17β-estradiol (Supplemental Figure 2).

In conclusion, we show 17β-estradiol modulates microtubule readouts in vitro, and in male PAB rats, 17β-estradiol mitigates microtubule remodeling and downstream pathogenic phenotypes. We identify a sexually divergent association between estrogen and RV microtubule/cardiomyocyte hypertrophy, as females do not increase RV tubulin abundance as robustly as males, and oophorectomy has minimal effects on tubulin regulation. However, exogenous 17β-estradiol reduces RV levels of α- and β-tubulin, combats RV cardiomyocyte hypertrophy, and enhances RV reserve in females. Thus, hindering microtubule remodeling could be another cardioprotective mechanism of 17β-estradiol, which adds to its well-documented advantageous signaling roles (4). However, we did not definitively parse out those differences. Interestingly, our female rodent results have human correlations, as higher serum estrogen levels from hormone replacement therapy are associated with superior right heart function in women (5).

Our data have important limitations, including confounding effects of alcohol as a vehicle in the treatment group. In addition, oophorectomy does not only cause loss of 17β-estradiol, but also causes loss of other sex hormones including dehydroepiandrosterone and progesterone, which may confound some of the estrogen-associated phenotypes. Finally, the improvements in RV function with exogenous estrogen are unlikely to be solely due to microtubule alterations, as estrogen modulates multiple aspects of cardiac biology (6).

See Supplemental Data for methods, additional data, funding information, and author contributions.

## Conflict of interest

SZP received personal fees from Merck. TL received personal fees and research funding from Allinaire, Inc. KWP obtained funding from Bayer and consulting fees from Merck.

## Funding support

This work is the result of NIH funding and is subject to the NIH Public Access Policy. Through acceptance of this federal funding, the NIH has been given a right to make the work publicly available in PubMed Central.

- T32AR007612, NIH (JBM).
- F31HL170585, National Heart, Lung, and Blood Institute (NHLBI) (JBM).
- 24POST1243617, American Heart Association (RSF).
- K08HL168166, NHLBI (SZP).
- 23CDA1049093, American Heart Association (SZP).
- 5R01HL144727, NHLBI (TL).
- 7I01 BX002042, Veterans Affairs (TL).
- 1P01HL158507, NIH (TL).
- Borstein Family Foundation (TL).
- R01HL158795, NHLBI (KWP).
- R01HL162927, NHLBI (KWP).

## Supplementary Material

Supplemental data

Unedited blot and gel images

Supporting data values

## Figures and Tables

**Figure 1:**
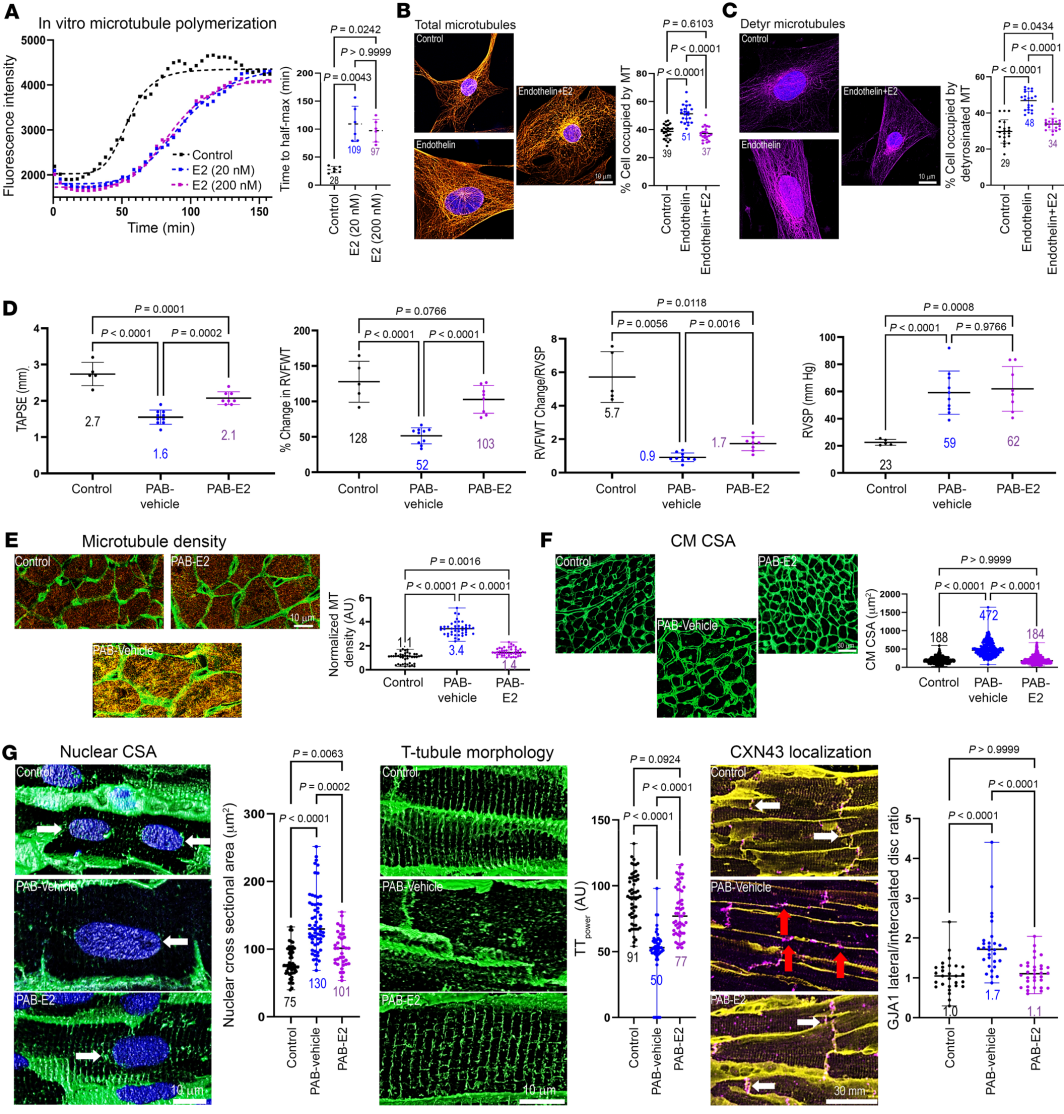
17β-Estradiol limits pathological microtubule stabilization and improves cardiac function in pulmonary artery–banded rats. (**A**) Microtubule polymerization kinetics in the presence or absence of 17β-estradiol (E2). Quantification of time to half max in the presence of ethanol vehicle, 20 nM 17β-estradiol, and 200 nM 17β-estradiol (E2). Representative confocal micrographs of iPSC-CMs stained with β-tubulin (orange, **B**), detyrosinated β-tubulin (purple, **C**), and DAPI (blue). Quantification of total microtubule (**B**) and detyrosinated microtubule (**C**) density. (**D**) E2 treatment increased RV function and estimated RV–pulmonary artery coupling but did not reduce right ventricular systolic pressure. RVFWT, RV free wall thickening. (**E**) Wheat germ agglutinin (WGA) (green) and microtubule staining (orange) of RV free wall sections from control, PAB-Vehicle, and PAB-E2 RV sections with quantification of relative microtubule fluorescence intensity. (**F**) WGA (green) delineated cardiomyocyte size. Values represent median area (black, blue, and purple). (**G**) 17β-Estradiol reduced nuclear size (white arrows), restored the normal striated t-tubule architecture in RV sections, and prevented lateralization of connexin-43 (purple) in the RV (red arrows: lateralized connexin-43, white arrows: connexin-43 at intercalated disc). Data are depicted as mean ± SD if normally distributed or median ± range if distribution was not normal. *P* values determined by 1-way ANOVA with Tukey’s multiple comparisons test or Brown-Forsythe ANOVA and Dunnett’s T3 multiple comparisons test.

## Version 1 05/07/2026

In-Press Preview

## Version 2 07/01/2026

Electronic publication
