# Supplementary material for: 17β-Estradiol counteracts pathological microtubule remodeling to enhance right ventricular function in preclinical models
Source: J Clin Invest. 2026 May 7;136(13):e201385. doi: 10.1172/JCI201385 (PMC13318100; doi:10.1172/JCI201385)
Supplement: Supplemental data [file jci-136-201385-s115.pdf]

Supplementary Data:

- Methods
- Reagents
- Author Contributions
- Funding Information
- Supplemental Figure 1
- Supplemental Figure 2

Methods

Sex as a biological variable: Our study examined male and female animals, and sex-dimorphic effects are reported.

In vitro microtubule polymerization assay: A fluorescent tubulin kit (Cytoskeleton:BK011P) was used to quantify *in vitro* microtubule polymerization rates in the presence of ethanol vehicle (*n*=6 replicates) or 17β-estradiol (Sigma-Aldrich, 20 nM (*n*=6 replicates) and 200 nM dissolved in ethanol (*n*=6 replicates).

Human induced pluripotent stem cells cardiomyocyte culture: Control iPSC (AICS-0011, Allen Institute) were differentiated into cardiomyocytes (iPSC-CM) as recommended by Allen Institute and as we have previously performed. All cells were beating before being subjected to chemical stress. To induce stress, iPSC-CMs were treated with endothelin (100nM, Tocris 1160, *n*=22) or endothelin+17β-estradiol (100 nM endothelin, 100 nM 17β-estradiol (E2), *n*=22) overnight and compared to control cells given vehicle (*n*=22-23).

iPSC-CM microtubule density assessment: iPSC-CMs were grown in chamber slides and then fixed with 4% paraformaldehyde for 10 minutes and then lysed with 1% Triton X100 in PBS for five minutes. Slides were then washed/blocked and incubated with primary antibodies to β-tubulin or detyrosinated α-tubulin at 4^0 overnight. Cells were washed with PBS and then incubated with Alexa-568 secondary antibody in PBS for 30 minutes at 37^0C. Cells were washed with PBS and then mounted in ProLong Glass with NucBlue Antifade Mountant. To determine microtubule density, z-stacks of cells were imaged on a Zeiss LSM900 Airyscan 2.0 microscope and the cytoplasmic portion of the cell on the opposite side of the microtubule aster was cropped by KWP. Images were then converted to 8-bit images and then the fraction of the cropped cell that was occupied by total microtubules or detyrosinated α-tubulin were calculated in FIJI using threshold function. All images were blindly analyzed by RAM.

Pulmonary artery banded rats and treatment: Adult male Sprague Dawley rats (Charles Rivers Laboratories) were subjected to pulmonary artery banding (PAB, 18-gauge needle) by the University of Minnesota Experimental Surgical Services. Two-weeks post banding, rats were randomly allocated to receive daily E2 treatment (0.2 mg/kg PAB-E2, *n*=10) or ethanol vehicle (PAB-Vehicle, *n*=10) via daily intraperitoneal injection for two weeks. There were compared to 5 control rats.

Confocal Microscopy of RV sections: Formalin sections of RV free walls were subjected to heat-mediated antigen retrieval (Reveal Decloaker, Biocare Medical), stained with primary antibodies to β-tubulin (*n*=41-45 total cells per group, from three different animals per group), desmin (*n*=65-98 total cells per group from three different animals per group), connexin-43 (*n*=28-32 total areas per group from three different animals per group), and then counterstained with AlexaFluor-568 anti-rabbit secondary antibody, Wheat Germ Agglutinin (WGA) AlexaFluor-488, and mounted. Confocal micrographs were collected on a Zeiss LSM900 Airyscan 2.0 microscope. Confocal images were blindly analyzed by RAM or SEP using FIJI.

Quantification of microtubule density in RV sections: Each day, one control, one PAB-Veh, and one PAB-E2 section was stained with β-tubulin antibody. Fluorescent intensity per area was determined for 15-20 cells per section per animal. The average value of the cells in controls was normalized to 1, and then the average

values from the PAB-Veh and PAB-E2 were compared to control. This process was repeated for over three days. This analysis was blindly performed by RAM.

Quantification of connexin-43 intercalated disc to lateral membrane ratio: Connexin-43 stained longitudinal sections were used to measure the intercalated disc to lateral membrane ratio. The relative fluorescent intensity of the intercalated disc and lateral membrane relative to area were determined blindly by RAM. The average value of the cells in controls (*n*=28 total areas was normalized to 1, and then the average values from the PAB-Veh (*n*=30) and PAB-E2 (*n*=32) were compared to control. The mean value of each animal was used as a single data point, and we evaluated 15-20 ratios per animal to get the single data point.

Quantification of cardiomyocyte cross-sectional area: WGA stained RV cross sections were used to quantify cross-sectional area. RV cardiomyocyte area of 25-100 cardiomyocytes per section per animal was blindly determined using FIJI by RAM and SEP. Total cardiomyocytes analyzed from male control (n=, PAB-Vehicle, PAB-E2, female control,

Quantification of nuclear cross-sectional area: DAPI and WGA stained RV longitudinal sections were used to quantify RV cardiomyocyte nuclear cross-sectional area. Nuclei area of at least 10 cardiomyocyte nuclei per section was blindly by RAM determined using FIJI. The number of nuclei analyzed were as follows: control male: *n*=51, PAB-Vehicle: *n*=60, PAB-E2: *n*=36 from three different animals per group. For female animals, four animals per group were analyzed with the following numbers: control: *n*=124, MCT: *n*=110, MCT-OVX: *n*=108, MCT-OVX-E2: *n*=104.

Quantification of t-tubule and desmin regularity: TTorg plugin in FIJI was used to quantify t-tubule and desmin regularity in RV longitudinal sections. 15-20 cells per section were scored and the average or median (depending on normality of distribution of values) value of each section was used as a single data point per animal. The number of cells analyzed for t-tubule morphology were as follows: control male: *n*=56, PAB-Vehicle: *n*=49, PAB-E2: *n*=53 from a total of three animals per group.

Rodent echocardiography: Echocardiography was performed using a Vevo2100 ultrasound system at the University of Minnesota. M-mode and 2-D modalities were used to measure tricuspid annular plane systolic excursion (TAPSE) and RV free wall thickness during end diastole and end systole.

Rodent closed-chest hemodynamics: Rats were anesthetized initially with 5% isoflurane induction and then maintained on 2-3% isoflurane. During the catheterization, rats were ventilated with the SomnoSuite small animal anesthesia system (Kent Scientific, Torrington, CT). A Scisense 1.9F catheter (Transonic Systems, Ithaca, NY) was advanced into the RV via the right internal jugular vein and right atria. RV pressures were continuously recorded using Transonic AV500 Pressure-Volume Measurement System and subsequently analyzed on LabScribe version 4 (iWorx Systems, Dover, NH). There were two PAB-E2 animals that were unable to get reliable pressure measurements during the hemodynamic assessments despite multiple repositioning attempts. These animals were excluded from the analysis.

Estimated RV-PA coupling: We estimated RV-PA coupling using the ratio of RV free wall thickening over right ventricular systolic pressure as has been performed in previous human studies.

Female monocrotaline rats and treatment: Female Sprague-Dawley rats (Charles Rivers Laboratories) were injected with monocrotaline (60 mg/kg) and then subjected to oophorectomy and oophorectomy plus 17β-estradiol replacement (75 μg/kg/day).

Immunoblots: Immunoblots of 25 μg of RV protein extracts were performed using the Odyssey Infrared Imaging system (Lincoln, NE) as previously described. Post transfer SDS-PAGE gels were stained with Coomassie brilliant blue (CBB) and imaged at the 700-nm wavelength on the Odyssey Imaging System with the band corresponding to the myosin heavy chain used as the loading control.

Langendorff Assessment: Hearts from female monocrotaline rats were rapidly isolated and perfused via the aorta at a constant pressure (60 mmHg) with oxygenated Krebs– Henseleit bicarbonate buffer (95% O2 + 5% CO2), in a temperature-controlled state (1, 2). Electrodes were placed at the aorta cannula and cardiac apex,

and hearts were paced at 350 beats/minute (Grass Instruments, S44 stimulator). Parameters of RV systolic function (developed pressure, maximum rate of pressure development [dP/dt max] were measured through a high-compliance fluid-filled balloon with a diameter of 3 mm inserted into the RV and connected to a pressure transducer. Each balloon was pretested for filling capacity before the beginning of experimentation. Parameters were recorded using a PowerLab data acquisition system (AD Instruments, Australia), and transducer calibration was performed before each experiment following the manufacturer's instructions. The Frank-Starling relationship was assessed in each heart by inflating the balloon with saline until the RV end-diastolic pressure (RVEDP) increased by 5 mmHg from 0 to 40 mmHg.

Statistical analyses: All analyses were performed using GraphPad Prism 10.1.1. When comparing the means of three groups, one-way analysis of variance (ANOVA) with Tukey post-hoc analysis was used when the variance was similar and the data were normally distributed. If the data were not normally distributed as assessed by the Shapiro-Wilk test, Kruskal-Wallis ANOVA with Dunn's post-hoc analysis was completed. If there was a normal distribution but unequal variance between groups, Brown-Forsythe and Welch ANOVA with Dunnett post-hoc analysis were completed. All ANOVA studies were one-way approaches.

Study Approval: Animal studies were approved by the University of Minnesota and the National Jewish Health Institutional Animal Care and Use Committees.

Data Availability: Data are available in the "Supporting data values" XLS file; or from the corresponding author upon request.

Reagents

**Primary Antibodies**			
Antigen	*Company*	*Catalogue Number*	*Dilution*
Alpha-tubulin	Millipore	05-829	1:250 (Western blot)
Beta-tubulin	Sigma-Aldrich	T4026	1:250 (Western blot)
Beta-tubulin	Abcam	AB6046	1:50 (Immunofluorescence of RV sections or iPSC-CMs)
Detyrosinated α-tubulin	Abcam	AB48389	1:50 (Immunofluorescence),1:500 (Western blot)
Connexin-43	Abcam	AB235585	1:50 (Immunofluorescence of RV sections)
Desmin	ProSci	46-777	1:50 (Immunofluorescence of RV sections)
**Secondary Antibodies and Fluorescent Stains**			
AlexaFluor-568 anti-rabbit secondary antibody	ThermoScientific	A-11036	1:500
Anti-mouse secondary infrared antibody	Li-COR	926-32210	1:5000
Anti-rabbit secondary infrared antibody	Li-COR	926-32211	1:5000
Wheat Germ Agglutinin (WGA) AlexaFluor-488	ThermoFisher	W21404	1:50

ProLong Glass with NucBlue Antifade Mountant	ThermoFisherScientific	P36985	N/A

Author Contributions: Performed experiments/analyzed data: RM, RF, MK, NV, JBM, LMH, JPC, SEP, SZP, KWP conceived and designed the study, performed experiments, analyzed data, TL and KWP wrote and edited the manuscript.

Funding Information:

1.T32AR007612, NIH (JBM)

2. F31HL170585, NHLBI (JBM)

3. 24POST1243617, American Heart Association (RSF)

4. K08HL168166, NHLBI (SZP)

5. 23CDA1049093, American Heart Association (SZP)

6. 5R01HL144727, NHLBI (TL)

7. 7I01 BX002042, Veteran Affairs (TL)

8. 1P01HL158507, NIH (TL)

9. Borstein Family Foundation (TL)

10. R01HL158795, NHLBI (KWP)

11. R01HL162927, NHLBI (KWP)

Supplemental Figure 1: Confocal micrographs of RV sections stained with WGA (purple) and desmin (green). Desmin localization patterns were similar in all three experimental groups. *p*-values determined by one-way ANOVA with Tukey's multiple comparison.

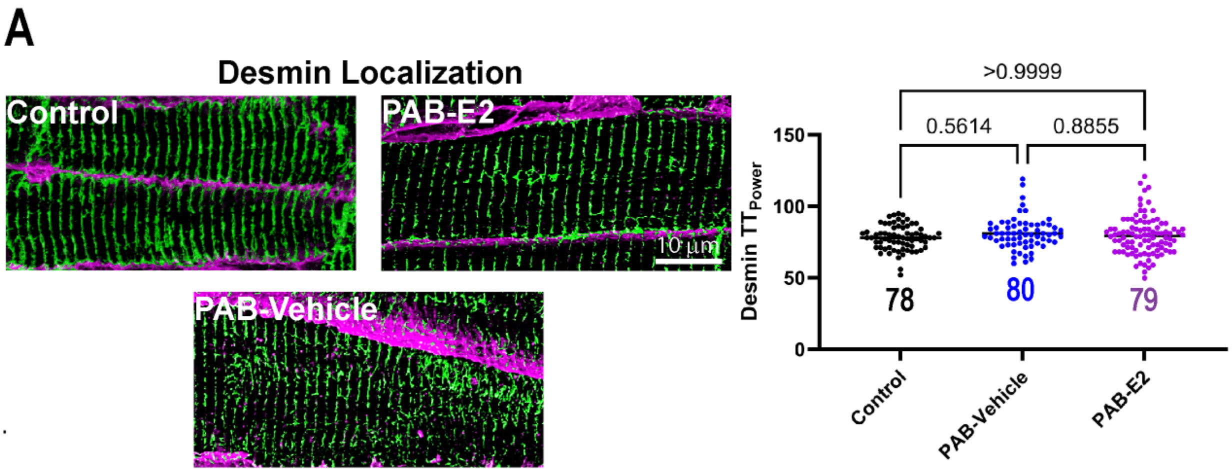

Supplemental Figure 2: 17β-estradiol modulated cardiomyocyte hypertrophy and Langendorff-based assessment of RV contractile reserve in female MCT rats: (A) Representation of groups evaluated to examine the effects of estrogen on RV microtubule remodeling in females (Left). Western blots from RV extracts examining tubulin isoforms (Right). Exogenous estrogen reduced α- and β-tubulin abundances as compared to MCT females. (J) Representative micrographs stained with WGA to outline cardiomyocytes and subsequent quantification of cardiomyocyte cross sectional area. *p*-values determined by one-way ANOVA with Tukey's multiple comparison for Western blot quantification and Kruskal-Wallis test with multiple comparisons for cardiomyocyte cross sectional area.

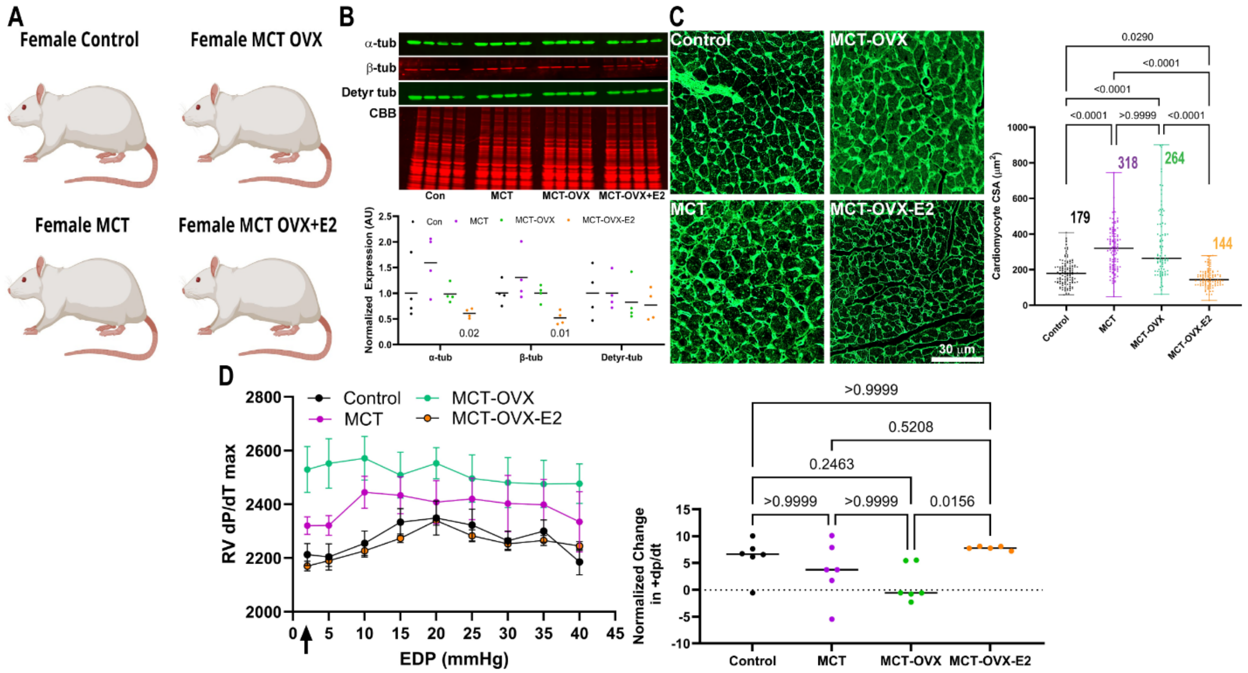

Raw Blots Used in Supplemental Figure 2

Alpha Tubulin
MW: 50 kDa
1:250

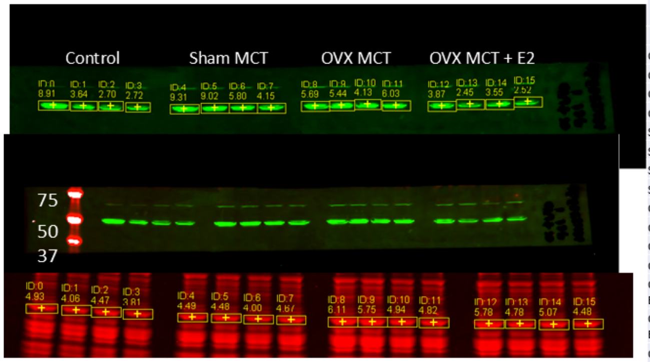

	Alpha tubulin	Normalizing band	Ratio	Control average	Percent control average	Percent control average (%)	Fold of control
Control 1	8.91	4.93	1.81	1.01	1.80	179.75	1.00
Control 2	3.64	4.06	0.90		0.89	89.17	
Control 3	2.70	4.47	0.60		0.60	60.08	
Control 4	2.72	3.81	0.71		0.71	71.00	
Sham MCT	9.31	4.49	2.07		2.06	206.23	1.60
Sham MCT	9.02	4.48	2.01		2.00	200.25	
Sham MCT	5.80	4.00	1.45		1.44	144.21	
Sham MCT	4.15	4.67	0.89		0.88	88.38	
OVX MCT	5.69	6.11	0.93		0.93	92.62	0.99
OVX MCT	5.44	5.75	0.95		0.94	94.10	
OVX MCT	4.13	4.94	0.84		0.83	83.15	
OVX MCT	6.03	4.82	1.25		1.24	124.43	
OVX MCT + E2	3.87	5.78	0.67		0.67	66.59	0.61
OVX MCT + E2	2.45	4.78	0.51		0.51	50.98	
OVX MCT + E2	3.55	5.07	0.70		0.70	69.64	
OVX MCT + E2	2.52	4.48	0.56		0.56	55.95	

Beta Tubulin
MW: 50 kDa
1:250

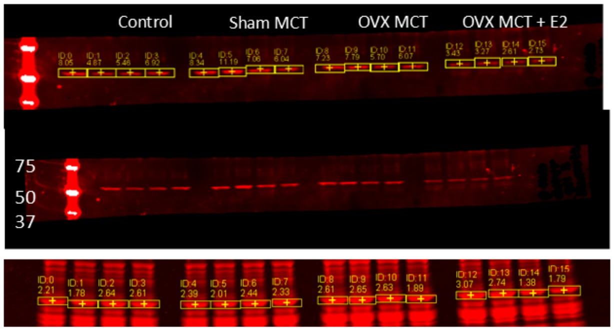

	Beta Tubulin	Normalizing band	Ratio	Control average	Percent control average	Percent control average (%)	Fold of control
Control 1	8.05	2.21	3.64	2.77	1.31	131.29	1.00
Control 2	4.87	1.78	2.74		0.99	98.61	
Control 3	5.46	2.64	2.07		0.75	74.54	
Control 4	6.92	2.61	2.65		0.96	95.56	
Sham MCT	8.34	2.39	3.49		1.26	125.77	1.31
Sham MCT	11.19	2.01	5.57		2.01	200.65	
Sham MCT	7.06	2.44	2.89		1.04	104.29	
Sham MCT	6.04	2.33	2.59		0.93	93.43	
OVX MCT	7.23	2.61	2.77		1.00	99.84	1.00
OVX MCT	7.79	2.65	2.94		1.06	105.95	
OVX MCT	5.70	2.63	2.17		0.78	78.11	
OVX MCT	6.07	1.89	3.21		1.16	115.76	
OVX MCT + E2	3.43	3.07	1.12		0.40	40.27	0.52
OVX MCT + E2	3.27	2.74	1.19		0.43	43.01	
OVX MCT + E2	2.61	1.38	1.89		0.68	68.17	
OVX MCT + E2	2.83	1.79	1.58		0.57	56.98	

Detyrosinated Tubulin
MW: 50 kDa
1:250

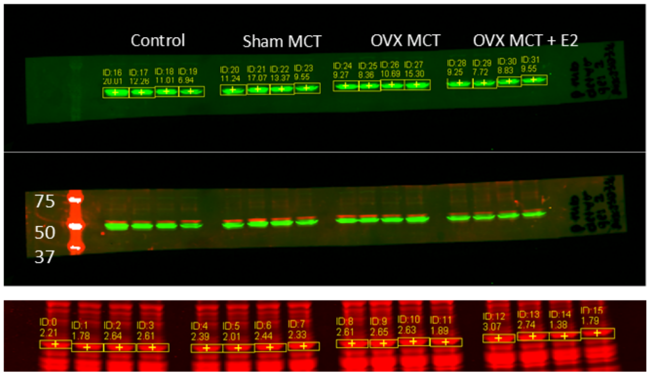

	Detyrosinated tubulin	Normalizing band	Ratio	Control average	Percent control average	Percent control average (%)	Fold of control
Control 1	20.01	2.21	9.05	5.69	1.59	159.05	1.00
Control 2	12.26	1.78	6.89		1.21	120.99	
Control 3	11.01	2.64	4.17		0.73	73.26	
Control 4	6.94	2.61	2.66		0.47	46.71	
Sham MCT	11.24	2.39	4.70		0.83	82.61	1.00
Sham MCT	17.07	2.01	8.49		1.49	149.18	
Sham MCT	13.37	2.44	5.48		0.96	96.25	
Sham MCT	9.55	2.33	4.10		0.72	72.00	
OVX MCT	9.27	2.61	3.55		0.62	62.39	0.83
OVX MCT	8.36	2.65	3.15		0.55	55.42	
OVX MCT	10.69	2.63	4.06		0.71	71.40	
OVX MCT	15.30	1.89	8.10		1.42	142.20	
OVX MCT + E2	9.25	3.07	3.01		0.53	52.93	0.77
OVX MCT + E2	7.72	2.74	2.82		0.49	49.49	
OVX MCT + E2	8.83	1.38	6.40		1.12	112.40	
OVX MCT + E2	9.55	1.79	5.34		0.94	93.72	

Supplemental References

1. Watts JA, Zagorski J, Gellar MA, Stevinson BG, and Kline JA. Cardiac inflammation contributes to right ventricular dysfunction following experimental pulmonary embolism in rats. J Mol Cell Cardiol. 2006;41(2):296-307.

2. Neto-Neves EM, Frump AL, Vayl A, Kline JA, and Lahm T. Isolated heart model demonstrates evidence of contractile and diastolic dysfunction in right ventricles from rats with sugen/hypoxia-induced pulmonary hypertension. Physiol Rep. 2017;5(19).
