# Supplementary material for: 17β-Estradiol counteracts pathological microtubule remodeling to enhance right ventricular function in preclinical models
Source: J Clin Invest. 2026 May 7;136(13):e201385. doi: 10.1172/JCI201385 (PMC13318100; doi:10.1172/JCI201385)
Supplement: Unedited blot and gel images [file jci-136-201385-s116.pdf]

Raw Blots Used in Supplemental Figure 2

Alpha Tubulin
MW: 50 kDa
1:250

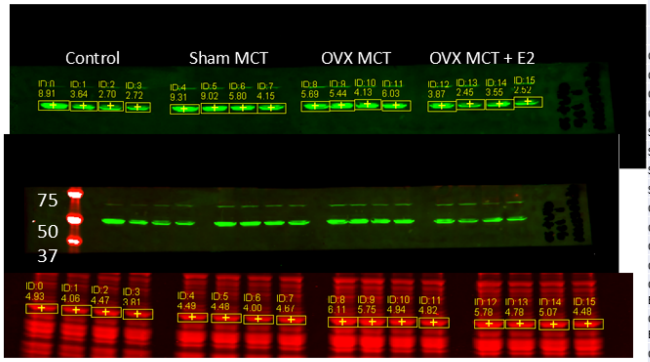

	Alpha tubulin	Normalizing band	Ratio	Control average	Percent control average	Percent control average (%)	Fold of control
Control 1	8.91	4.93	1.81	1.01	1.80	179.75	1.00
Control 2	3.64	4.06	0.90		0.89	89.17	
Control 3	2.70	4.47	0.60		0.60	60.08	
Control 4	2.72	3.81	0.71		0.71	71.00	
Sham MCT	9.31	4.49	2.07		2.06	206.23	1.60
Sham MCT	9.02	4.48	2.01		2.00	200.25	
Sham MCT	5.80	4.00	1.45		1.44	144.21	
Sham MCT	4.15	4.67	0.89		0.88	88.38	
OVX MCT	5.69	6.11	0.93		0.93	92.62	0.99
OVX MCT	5.44	5.75	0.95		0.94	94.10	
OVX MCT	4.13	4.94	0.84		0.83	83.15	
OVX MCT	6.03	4.82	1.25		1.24	124.43	
OVX MCT + E2	3.87	5.78	0.67		0.67	66.59	0.61
OVX MCT + E2	2.45	4.78	0.51		0.51	50.98	
OVX MCT + E2	3.55	5.07	0.70		0.70	69.64	
OVX MCT + E2	2.52	4.48	0.56		0.56	55.95	

Beta Tubulin
MW: 50 kDa
1:250

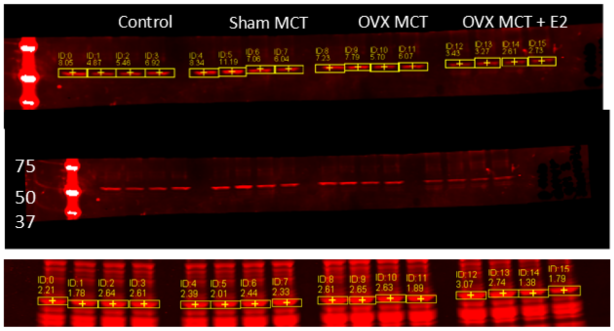

	Beta Tubulin	Normalizing band	Ratio	Control average	Percent control average	Percent control average (%)	Fold of control
Control 1	8.05	2.21	3.64	2.77	1.31	131.29	1.00
Control 2	4.87	1.78	2.74		0.99	98.61	
Control 3	5.46	2.64	2.07		0.75	74.54	
Control 4	6.92	2.61	2.65		0.96	95.56	
Sham MCT	8.34	2.39	3.49		1.26	125.77	1.31
Sham MCT	11.19	2.01	5.57		2.01	200.65	
Sham MCT	7.06	2.44	2.89		1.04	104.29	
Sham MCT	6.04	2.33	2.59		0.93	93.43	
OVX MCT	7.23	2.61	2.77		1.00	99.84	1.00
OVX MCT	7.79	2.65	2.94		1.06	105.95	
OVX MCT	5.70	2.63	2.17		0.78	78.11	
OVX MCT	6.07	1.89	3.21		1.16	115.76	
OVX MCT + E2	3.43	3.07	1.12		0.40	40.27	0.52
OVX MCT + E2	3.27	2.74	1.19		0.43	43.01	
OVX MCT + E2	2.61	1.38	1.89		0.68	68.17	
OVX MCT + E2	2.83	1.79	1.58		0.57	56.98	

Detyrosinated Tubulin
MW: 50 kDa
1:250

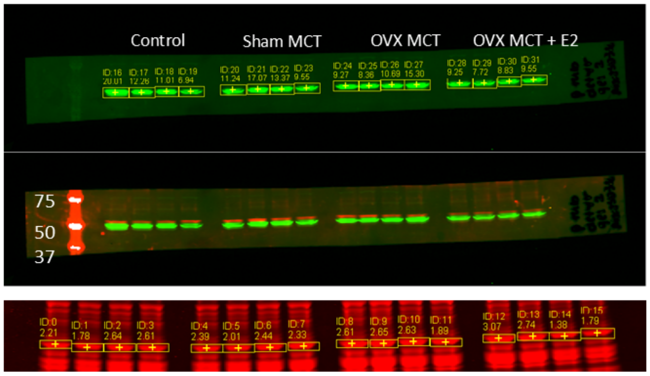

	Detyrosinated tubulin	Normalizing band	Ratio	Control average	Percent control average	Percent control average (%)	Fold of control
Control 1	20.01	2.21	9.05	5.69	1.59	159.05	1.00
Control 2	12.26	1.78	6.89		1.21	120.99	
Control 3	11.01	2.64	4.17		0.73	73.26	
Control 4	6.94	2.61	2.66		0.47	46.71	
Sham MCT	11.24	2.39	4.70		0.83	82.61	1.00
Sham MCT	17.07	2.01	8.49		1.49	149.18	
Sham MCT	13.37	2.44	5.48		0.96	96.25	
Sham MCT	9.55	2.33	4.10		0.72	72.00	
OVX MCT	9.27	2.61	3.55		0.62	62.39	0.83
OVX MCT	8.36	2.65	3.15		0.55	55.42	
OVX MCT	10.69	2.63	4.06		0.71	71.40	
OVX MCT	15.30	1.89	8.10		1.42	142.20	
OVX MCT + E2	9.25	3.07	3.01		0.53	52.93	0.77
OVX MCT + E2	7.72	2.74	2.82		0.49	49.49	
OVX MCT + E2	8.83	1.38	6.40		1.12	112.40	
OVX MCT + E2	9.55	1.79	5.34		0.94	93.72	
